# Detection of viral sequence fragments of HIV-1 subfamilies yet unknown

**DOI:** 10.1186/1471-2105-12-93

**Published:** 2011-04-11

**Authors:** Thomas Unterthiner, Anne-Kathrin Schultz, Jan Bulla, Burkhard Morgenstern, Mario Stanke, Ingo Bulla

**Affiliations:** 1 Institute of Microbiology and Genetics, University of Göttingen, Goldschmidtstr. 1, 37077 Göttingen, Germany; 2 LMNO, Université de Caen, CNRS UMR 6139, 14032 Caen Cedex, France; 3 Institut für Mathematik und Informatik, Walther-Rathenau-Straße 47, 17487 Greifswald, Germany

## Abstract

**Background:**

Methods of determining whether or not any particular HIV-1 sequence stems - completely or in part - from some unknown HIV-1 subtype are important for the design of vaccines and molecular detection systems, as well as for epidemiological monitoring. Nevertheless, a single algorithm only, the Branching Index (BI), has been developed for this task so far. Moving along the genome of a query sequence in a sliding window, the BI computes a ratio quantifying how closely the query sequence clusters with a subtype clade. In its current version, however, the BI does not provide predicted boundaries of unknown fragments.

**Results:**

We have developed *Unknown Subtype Finder *(USF), an algorithm based on a probabilistic model, which automatically determines which parts of an input sequence originate from a subtype yet unknown. The underlying model is based on a simple profile hidden Markov model (pHMM) for each *known *subtype and an additional pHMM for an *unknown *subtype. The emission probabilities of the latter are estimated using the emission frequencies of the known subtypes by means of a (position-wise) probabilistic model for the emergence of new subtypes. We have applied USF to SIV and HIV-1 sequences formerly classified as having emerged from an unknown subtype. Moreover, we have evaluated its performance on artificial HIV-1 recombinants and non-recombinant HIV-1 sequences. The results have been compared with the corresponding results of the BI.

**Conclusions:**

Our results demonstrate that USF is suitable for detecting segments in HIV-1 sequences stemming from yet unknown subtypes. Comparing USF with the BI shows that our algorithm performs as good as the BI or better.

## Background

An accurate and reliable classification of viral sequences data for human immunodeficiency virus-1 (HIV-1) and some other viruses of interest is important for epidemiological studies. It facilitates the understanding of the influence of genetic diversity on host immune response and provides therapeutic decision support [1-3]. As HIV-1 is, however, one of the genetically most variable viruses and genomic recombinations are frequent in HIV-1 [4], the task of classifying corresponding viral sequence data is a challenging one.

HIV-1 is classified into three main phylogenetic groups (M, N, and O), introduced into humans by separate zoonotic events (all stemming from simian immunodeficiency viruses (SIVs) in chimpanzees [5]. The M group is responsible for the HIV pandemic, and it is divided into nine subtypes, with subtype A and F being subdivided into subsubtypes [6]. Inter-subtype recombination occurs very frequently among HIV-1 subtypes [7]: So far, 48 circulating recombinant forms have been reported [8].

Up to now, about fifty tools for classification of HIV genomes, recognition of recombinants, and breakpoint detection have been developed. Examples are the REGA HIV-1 Subtyping Tool [9], the Recombinant Identification Program (RIP) [10], the jumping profile Hidden Markov Model (jpHMM) [11,12], the Recco [13], and the oligonucleotide-based method introduced in [14]. Nevertheless, to our knowledge, however, only one algorithm, called the Branching Index (BI), has been developed for deciding whether an HIV-1 sequence in question stems - completely or in part - from a subtype still unknown [15,16]. Notice that it is impossible to deduce unknown sequence segments using an existing subtype classification method, based on a probabilistic model such as jpHMM, and to identify regions of low a posteriori probabilities for all of the well known subfamilies (see paragraph 'Discussion and conclusions - Miscellaneous').

In view of the large and rapidly growing quantity of sequence data, the need for a fully automatic tool for pinning down boundaries of unknown fragments is increasing. Since the BI is based on a sliding window approach, it only provides a visualization of the breakpoint positions, but no report of their exact position. We have addressed this problem by developing a model-based algorithm, which automatically detects those boundaries by taking a multiple sequence alignment (MSA) grouped into subfamilies as a basis.

A comparison of our algorithm with the BI, regarding scope and performance, is carried out in the section 'Results - Comparison'.

## Methods

The main input into our algorithm consists of i) an MSA representing the known sequences, with its sequences grouped into subfamilies, ii) a query sequence, iii) a classification of the query sequence with respect to the subfamilies (i.e. each position of the query sequence has to be assigned to a subfamily from the MSA) as main input.

**Figure 1:**
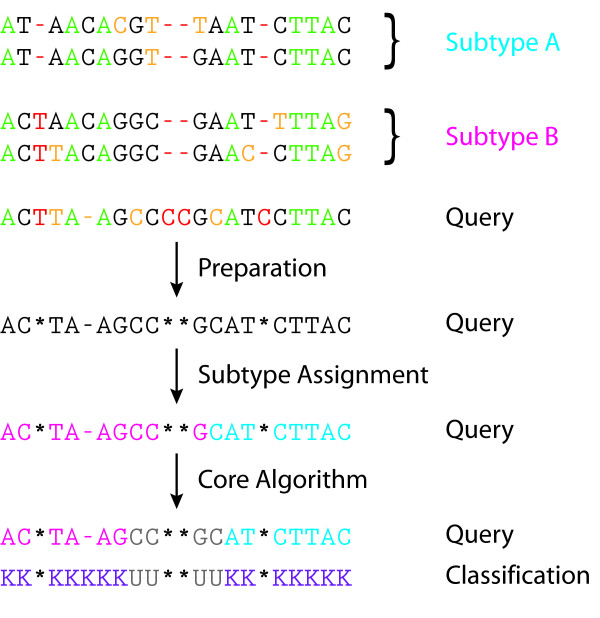
**Method outline**. Outline of the steps of the method, assuming 2 subtypes composed each of 2 sequences. The following color code for columns and nucleotides, respectively, is used in the topmost part: green - completely conserved columns (with respect to all subtype sequences and the query sequence), red - columns removed due to insertion by the query sequence or too much gaps in the alignment, yellow - minority nucleotides in the column. The part of the sequence coloured in gray (at the bottom) indicates a subtype yet unknown. The last row gives the classification of the query sequence into known and unknown positions.

We use jpHMM in order to obtain the subfamily-wise classification [17]. For each position of the sequence in question, the algorithm then provides a mapping which determines whether the assignment to the subfamily is justified or whether it has to be classified as belonging to a subfamily yet unknown. In the first case, we refer to the position as 'known' (sometimes abbreviated by K), in the second one as 'unknown' (sometimes abbreviated by U). We shall refer to the mapping as the 'U/K-classification'. The work flow of the core algorithm and the preparatory step (described in the next subsection) is illustrated in Figure 1. The subfamily assigned to position *i *of the sequence under discussion is denoted by *F_i_*.

### Preparatory step

Before the core algorithm is carried out, we take a preparatory step, allowing for the input of unaligned query sequences. More precisely, we align the query sequence to the given alignment with ClustalW [18], and remove columns which i) constitute insertions in the alignment by the query sequence, or ii) contain too many gaps in the alignment (we use a threshold of 10% gaps).

### Core algorithm

The main idea of our algorithm (in the following referred to as Unknown Subtype Finder or USF) is that of constructing two simple pHMMs (allowing neither deletions nor insertions): The first pHMM models the sequence of predicted subtypes *F_i _*for each position (in the following pHMM K) and the second pHMM models an unknown subtype (in the following pHMM U). That is, for the example given in Figure 1, the first ten positions of pHMM K are modelled on the basis of the nucleotide frequencies of Subtype B at those positions and the last eight positions on the basis of the frequencies of Subtype A. In addition to the transitions within these two pHMMs, we allow for jumps between them.

#### pHMMs K and U

pHMMs are widely used for modelling nucleotide and protein sequence families for the purpose of database searching (see [19,20]). In particular, they are used to model the position-wise nucleotide distribution in an MSA. Standard pHMMs also allow for the modelling of insertions and deletions in the query sequence. But we do not use insertion or deletion states, as the sequences are already aligned (The high conservation of HIV-1 sequences allows for this approach). Hence, except for the initial and final states, our pHMMs are composed of so-called match states only. For decoding the most probable path through our model, we use the Viterbi algorithm [21].

We model pHMM K in the conventional way: For each position *i *in the alignment, we model the emission probabilities  of the *i*-th state of the pHMM K on the basis of the nucleotide frequencies of *F_i_*. To this end, choosing a Bayesian approach to model the emission frequencies, we assume that the a priori distribution of  is a Dirichlet distribution (see [22]), with parameter  (estimated in [17]). The parameter may be interpreted as pseudo counts which are added to the nucleotide frequencies. The emission probabilities then are the corresponding relative frequencies of these modified nucleotide frequencies.

For pHMM U, we have to choose another approach, as the empirical nucleotide frequencies of an unknown subtype are not available. Hence, we try to deduce reasonable emission probabilities of an unknown subtype on the basis of the nucleotide frequencies of the known subtypes. For more details, see the paragraph 'Emission probabilities of pHMM U' in this subsection.

#### Jumps between pHMMs

**Figure 2:**
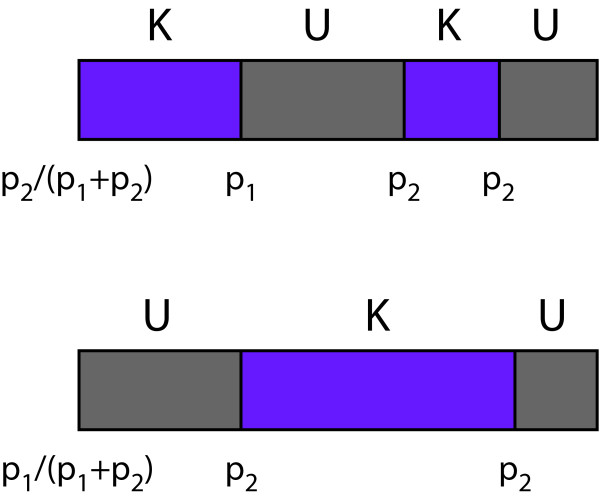
**Jumping probabilities**. Jumping probabilities for two examples of U/K-classifications. Under the breakpoints, the jumping probabilities are given.

As in the jpHMM, we allow for jumps between the pHMMs K and U. If a given path contains a jump, that jump represents a breakpoint between a known and an unknown segment. In our model, we distinguish two kinds of jumps (passing from left to right): (i) jumps from K to U with the path not having entered any state of pHMM U up to the current position, and (ii) all other jumps between K and U (see Figure 2 for examples for the determination of the jump probabilities). The probability of the first type of jumps is denoted by *p*_1_, the probability of the second type by *p*_2_. By modelling jumps in this way, we account for the fact that HIV-1 recombination events usually imply the occurrence of multiple breakpoints (cf. [8]). That is, traversing an HIV-1 genome from left to right, it is much more probable to revisit a particular subtype than it is to visit it for the first time ever. So, a realistic model should allow for choosing *p*_1 _≪ *p*_2_. To cover the case where the first position is classified as unknown, a jump from the initial state to pHMM U is less probable than a jump to pHMM K by the factor *p*_2_/*p*_1_.

**Figure 3:**
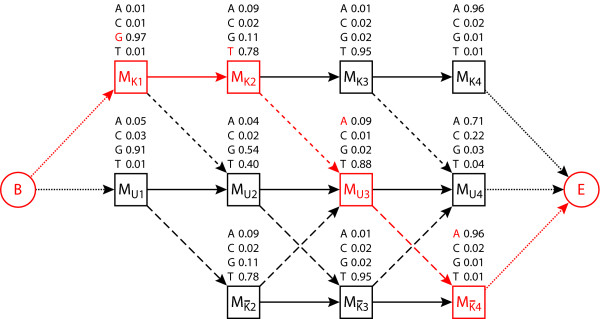
**Model**. The model underlying USF, illustrated by a toy example. The example uses an alignment and a query sequence of length 4. The query sequence is composed of the nucleotide sequence GTAA. The top row and bottom row of states each constitute a pHMM K, the middle one pHMM U. The top pHMM K models the situation of pHMM U not having been visited yet, the bottom one that of pHMM U having been visited already. Above and below, respectively, the states, their emission probabilities are given, with the nucleotide in the query sequence being marked red for the states in the Viterbi path. To the very left, resp. the very right, the initial, resp. the final state are situated. The short-dashed arrows represent transitions with probability *p*_2_, the long-dashed ones transitions with probability *p*_1_. The dotted arrows constitute transitions from and to special states (initial and final state). The Viterbi path is colored in red, with the first two positions and the last position of the query sequence being classified as 'known' and the third position as 'unknown'. Notice that the first state of the bottom pHMM K is missing since this pHMM can only be entered if pHMM U has been visited before.

In order to be able to model these two jump probabilities, we have to incorporate the pHMM K in our model twice: Both model states represent the assignment of a position to be known, with one of them being used if no position has been assigned as unknown so far, and the other being applied if some position has been assigned to pHMM U already. Figure 3 shows a toy example of our model.

#### Emission probabilities of pHMM U

In order to model the emission probabilities of pHMM U, we rely on the observation that for almost all sites for HIV-1 at least some of the subtypes share the same emission probabilities. In fact, for the majority of sites, it would be most plausible to assign equal emission probabilities to all subtypes. Neglecting the trivial case of all subtypes having the same emission probability assigned to, the phenomenon that some but not all of the subtypes show equal emission probabilities could be explained biologically as follows: If a site allows for more than one nucleotide to be present (i.e., if at least two alleles are observed), there are very few, discrete characteristics of the virus which determine the fitness of the virus, depending on the nucleotide present at the respective site. As the characteristics at a particular site are small in number and discrete, the number of corresponding nucleotide distributions is also small. To clarify that, let us assume that for a site *i *the dependence of the virus fitness on the nucleotide at site *i *is determined by a binary characteristics (values 0 and 1) of the virus. Then i) for the value 0, it might be that the virus can only survive if adenine is present at site *i *(leading to a nucleotide distribution where adenine has a probability very near to one), ii) for the value 1 the virus can survive if cytosine is present, with a significant disadvantage with respect to its fitness (leading to a nucleotide distribution where adenine has a probability of, say, about 90% and cytosine one about 10%). In the following we will call the different nucleotide distributions (resp. emission probabilities) at the site "sources". In the example just given there are two sources.

**Figure 4:**
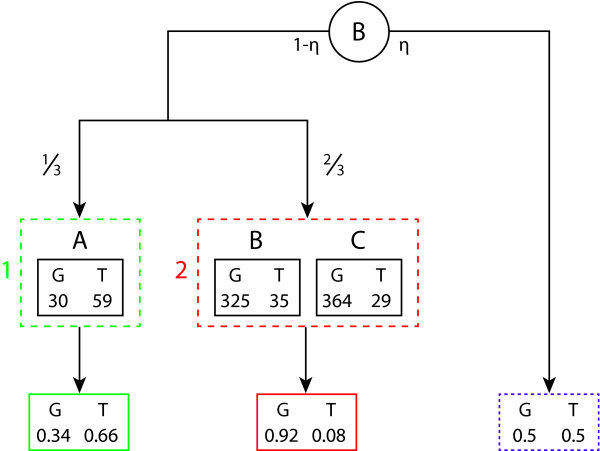
**Modelling of the emission probabilities of pHMM U**. The model used for the emission probabilities of pHMM U, illustrated by a toy example. Assumed are 3 subtypes (A, B, C), which are assigned to two sources: A to Source 1 (green), B and C to Source 2 (red). Only two nucleotides, G and T, are assumed to exist and  is set to (0.1, 0.1). With probability *η *the right-hand part of the model is chosen, and with probability 1-*η *the left-hand one. If the left-hand part is chosen, then with probability  Source 1 is then chosen, and with probability  Source 2. At the bottom, the generated emission probabilities are given for the different paths the model can take. In case the right-hand part is chosen, a Dirichlet distribution with parameter  is taken for the generation of the emission probabilities. The emission probabilities of pHMM U are estimated by averaging over all possible emission probabilities, weighting them with their respective probabilities. That is, assuming *η *= 0.05, we obtain  as estimate for the emission probabilities.

In view of such considerations, we model the emission probabilities of the subtypes jointly (see Table 1 for examples). Notice that a related approach was used in [23] for an automatic classification of protein sequences. The model for the emission probabilities of an unknown subtype is illustrated in Figure 4. It is composed of two parts: The part on the left refers to the case in which the unknown subtype is related to a group of known subtypes (or a single one) sharing the same emission probability at the respective site. The part on the right concerns the case of an unknown subtype with characteristics leading to emission probabilities (at the respective sites) yet unobserved (among the known subtypes).

**Table 1:** Examples of calculation of emission probabilities

**Pos**.	**Sub./Src**.	A		B		C		D		1		2		*3*	
	**Nucl**.	G	T	G	T	G	T	G	T	G	T	G	T	G	T
1	freq	89	0	360	0	393	0	3	0	846	0				
	p	0.9989	0.0011	0.9997	0.0003	0.9997	0.0003	0.969	0.031	0.9999	0.0001				

2	freq	65	24	**355**	**5**	**382**	**11**	**3**	**0**	65	24	**740**	**19**		
	p	0.73	0.27	0.986	0.014	0.972	0.028	0.969	0.031	0.73	0.27	0.975	0.025		

3	freq	30	59	**325**	**35**	**364**	**29**	*0*	*3*	30	59	**689**	**64**	*0*	*3*
	p	0.34	0.66	0.903	0.097	0.926	0.074	0.0031	0.969	0.34	0.66	0.915	0.085	0.0031	0.969

Simplified example of position- and subtype-wise nucleotide frequencies of HIV. For three sites the subtype-wise nucleotide frequencies for subtypes A, B, C, and D are given on the left side of the table. Below them the emission probabilities estimated on the basis of only on the frequencies of the respective subtypes (using ) are shown. The different typefaces (regular, bold, italic) indicate which subtypes should be jointly modelled (i.e. belong to the same source). On the right-hand side of the table, the nucleotide frequencies of the sources (i.e. the aggregated frequencies of the subtypes belonging to it) and the emission probabilities estimated on the basis of these frequencies are given (using the same ). For the sake of simplicity, only the nucleotides G and T are assumed to exist. Apart from this simplification and the restriction to 4 subtypes, the example is taken from actual HIV-1 sequences.

To construct the left-hand part of the model, we use a Bayesian approach to determine position-wise an optimal number of sources and how the subtypes should be assigned to the sources. For each source the emission probabilities are estimated on the basis of the emission frequencies of the subtypes assigned to the source. The probability, with which a source is chosen, is proportional to the number of subtypes assigned to it. The right-hand part is modelled by a Dirichlet distribution with the same value for the parameter  as in paragraph 'pHMMs K and U' of this subsection. We denote the a priori probability of a source involved, but yet unknown, by *η *≪ 1. The estimates of the emission probabilities of the unknown subtype are obtained by averaging over both parts of the model, i.e. we use the expectations corresponding to the emission probabilities under the model. The details of the estimation procedure are given in the next subsection.

### Details of pHMM U

#### Notation

Let 1,..., *S *be the subtype indices. If sources have been assigned to all subtypes, we speak of a source combination. The individual sources in a combination of *r *sources are indexed by 1,..., *r*. The space of all source combinations is denoted by Q, the source of subtype *i *by *q_i_*. For each source *j *of a source combination , we denote the subtypes assigned to source *j *by. That is, if *S *= 4 and the subtypes 1, 2, and 4 are assigned to Source 1, and the Subtype 3 to Source 2, we have *m*_1 _= 3, *m*_2 _= 1, , and  (Notice that *r *and the  are defined for a particular source combination, but that for the sake of readability we do not identify that source by an additional index, in case several sources are considered). The number of nucleotides, generally denoted by *N*, is equal to 5 in this case (We treat gaps as ordinary nucleotides). The nucleotide frequencies of subtype *i *at a fixed position of the genome are denoted by .

#### Prior probability of number of sources

We denote the probability of a given number of sources by . It is estimated as follows: We compile an alignment of all available HIV-1 sequences of complete length, classified as a pure subtype in the LANL HIV database (i.e. not being identified as recombinant or unknown). Hereby, we discard all sites at which the sequences of at least one subtype have only gaps. Then we determine, site-wise for each number of sources, the most probable source combination yielding the number of sources under consideration. For that we need the likelihood of , which is given by(1)

The probabilities on the right hand side of (1) can be calculated as described in the following. For the next step we restrict ourselves to the case that  for notational convenience and make use of the equations(2)

and(3)

as well as(4)

With *β*_1_,..., *β_N _*≥ 0. Here,  denotes the parameter of the Dirichlet distribution introduced in the paragraph 'Methods - Core algorithm - pHMMs K and U'. Thus, we obtain

Using (1) and the AIC (Akaike Information Criterion [24]), we deduce the most plausible source combination for each site and with that the most plausible number of sources. Estimating the *ρ_j _*as the empirical frequencies of the number of sources (considering all eligible sites), we obtain the values (*ρ_j_*)_*j *= 1,2,3 _= (0.85, 0.09, 0.06). For the sake of computational efficiency, we restrict the number of sources to values lower or equal to 3. Notice that the number of sources to which one can restrict the algorithm depends on the scale of the intersubtype variation of the virus genome at the informative sites of the genome.

#### Estimation of emission probabilities

Using

we deduce the most likely source combination. Then, for a given source combination , we can estimate the emission probability of a nucleotide *v *for a particular source (assuming, for notational convenience, that the source under consideration is composed of the subtypes 1,..., *m*) by(5)

Using (2) and (3), we get

Consequently, we can transform (5) into

Finally, by using (4) we obtain the simple formula

## Results

In this section, we present the results of i) the calibration of USF on a) artificial HIV-1 recombinants and b) non-recombinant HIV-1 sequences designated as having emerged from a known subtype, ii) the application of USF to a) SIV sequences and b) sequences designated as unknown in the LANL HIV database (in the following called "Subtype U" sequences), and iii) the comparison of USF and BI.

### Calibration

**Figure 5:**
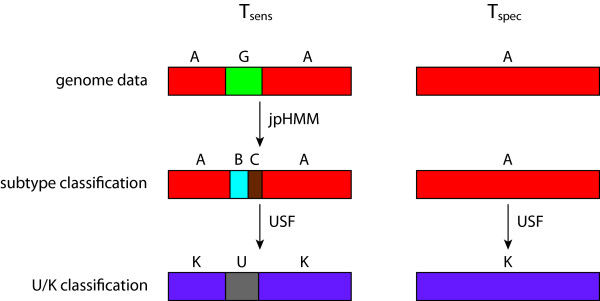
**Test setup**. Testing is performed on two sets of sequence data, *T_sens _*and *T_spec_*. For *T_sens _*artificial recombinants of two subtypes are used as genome data, for *T_spec _*pure sequences are taken. The sequences of *T_sens _*are classified subtype-wise with jpHMM, whereas the sequences of *T_spec _*are assigned their original subtype. Then, USF is applied to both sequence sets.

In order to calibrate USF and to investigate its behaviour in dependence of the choice of the parameters *η*, *p*_1 _and *p*_2_, we use two test settings, one of them suitable to assess the sensitivity of the algorithm, the other one the specificity. For the sensitivity, we remove one subtype from the MSA and consider it as unknown. Then we generate artificial recombinants of sequences from the "known" subtypes and the "unknown" subtype. For the specificity, we simply check whether sequences from the MSA are classified correctly. In both cases, we do not use the test data as training data for the emission probabilities of the HMMs. The testing setup is sketched in Figure 5. The MSA consists in all full-length HIV-1 Group M sequences, designated as stemming from a pure subtype in the LANL database, downloaded on 9th of July 2010.

#### Test data

More precisely, we generate the following two sets of test sequences: (i) A set *T_sens _*for measuring the sensitivity with respect to the ability of the algorithm to detect genome segments stemming from an unknown subtype, and (ii) a set *T_spec _*for measuring the specificity. The set *T_sens _*is composed of 229 sequences generated by taking a sequence from subtypes A-D and F-G and replacing a segment of this sequence by a segment of a sequence from some other subtype. We call the subtype of the major part of the genome the 'base subtype' and the subtype of the inserted part of the genome the 'insertion subtype'. A preliminary analysis shows that in case the subtypes H, J, or K have been assigned to the query sequence (or a part of it), USF is not suitable for a reliable detection of unclassifiable genome parts. Hence, for the role of a base subtype, those subtypes are excluded from our analysis. Nevertheless, segments of them may play the role of insertion subtypes. Segments of the subtypes B and D may not be combined, due to the small phylogenetic distance of those subtypes. Moreover, the replaced segments have a length of 1000 positions and their position has been chosen randomly. *T_spec _*is composed of 265 sequences sampled from the genome-length sequences being classified as subtype A-D or F-G in the LANL HIV database (50 for all subtypes except for the subtypes F and G, for which only 35 and 30, respectively, sequences were available). For *T_spec _*the sequences were assigned to the subtype they stem from according to their LANL HIV database designation. Therefore, if classified correctly, the complete sequence is classified as known. Any detected unknown regions are counted as false positives. For *T_sens _*we determine the subtype classification using the jpHMM, excluding the subtypes H, J, and K from the assignable subtypes.

#### Test results

**Figure 6:**
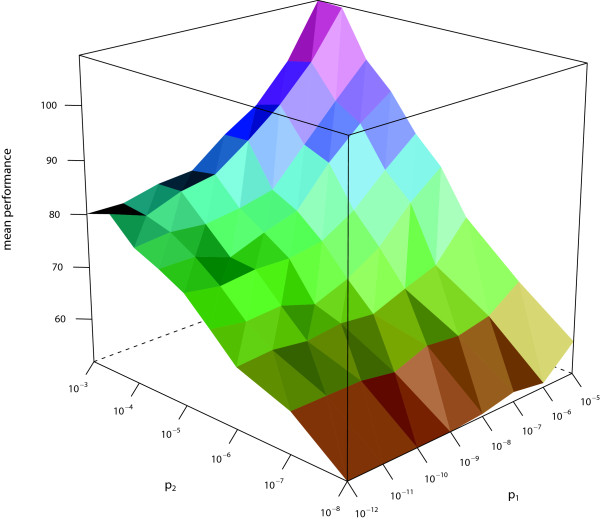
**Mean performance of *T_spec_***. The mean performance (measured in misclassified positions) of *T_spec _*in dependence of *p*_1 _and *p*_2 _(both scaled logarithmically).

**Figure 7:**
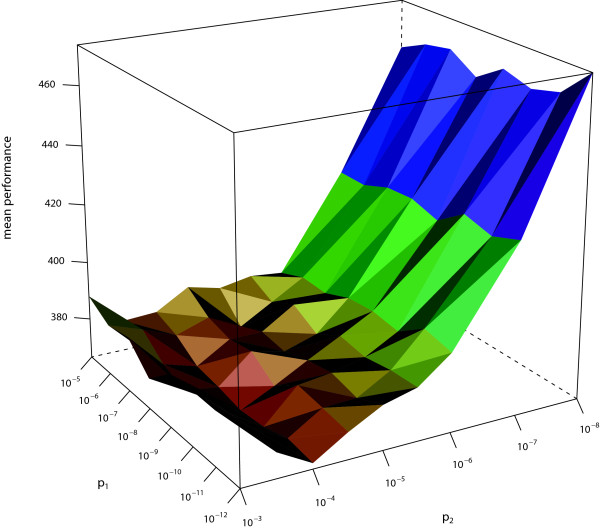
**Mean performance of *T_sens_***. The mean performance (measured in misclassified positions) of *T_sens _*in dependence of *p*_1 _and *p*_2 _(both scaled logarithmically).

We measure the performance by counting how many positions in a sequence have been misclassified. Setting *p*_1 _= 10^-7 ^and *p*_2 _= 10^-4 ^(which seem to be reasonable values, in view of our experience gathered when applying the jpHMM to HIV), we determine *η *= 0.05 as leading to the best tradeoff between sensitivity and specificity. With that choice of *η*, we evaluate the performance with respect to specificity and sensitivity on a grid for different choices of *p*_1 _and *p*_2 _(see Figure 6 and 7). From those data, we would recommend to choose *p*_1 _= 10^-9 ^and *p*_2 _= 10^-5^. In case a user has a different priority with respect to specificity and sensitivity, he can adapt the values to his purpose. To achieve a higher sensitivity or specificity, *p*_1 _and *p*_2 _have to be increased or decreased, respectively. Increasing *p*_1 _merely results in a higher probability of finding any Subtype U fragments in the query sequence at all, whereas increasing *p*_2 _also leads to a higher number of Subtype U fragments to be found.

**Figure 8:**
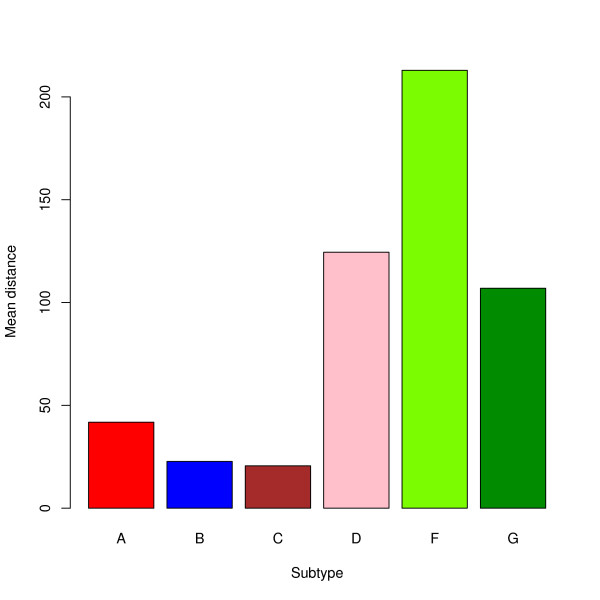
**Mean subtype-wise performance of *T_spec_***. The mean performance (measured in misclassified positions) of *T_spec _*for *p*_1 _= 10^-9 ^and *p*_2 _= 10^-5^, stratified by the assigned subtype.

In Figure 8, resp. 9, the performance of the algorithm for *T_spec_*, resp. *T_sens _*is displayed stratified by the assigned subtype *T_sens_*, resp. the subtypes used for generating the artificial recombinants. Among the 6 sequences from *T_spec_*, which yield the most misclassified positions, there are all 4 sequences of Subsubtype F2 and the sequence from Subsubtype F1, which cluster most closely to Subsubtype F2 in a phylogenetic tree (using FastTree [25] and FigTree [26]).

**Figure 9:**
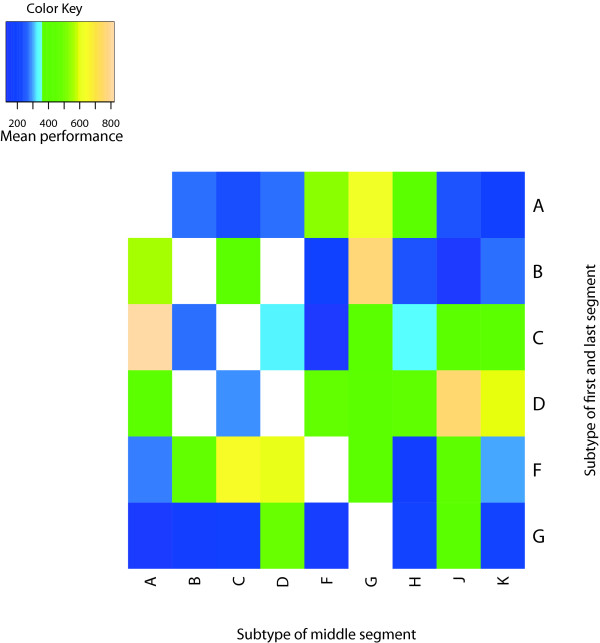
**Mean subtype-wise performance of *T_sens_***. Level plot of the mean performance (measured in misclassified positions) of *T_sens _*for *p*_1 _= 10^-9 ^and *p*_2 _= 10^-5^, stratified by the used subtypes. Different colors represent different levels of misclassification. White rectangles represent subtype pairs which were not used in the generation of *T_sens_*.

To facilitate the testing technically, we restrict our analysis to the positions 808 to 8781 with respect to HXB [27]. Covering this part of the genome, we analyse the performance of USF in relatively conserved regions, as well as highly variable ones and we do not have to cope with the low number of sequences available for covering the boundary parts of the genome.

#### Theoretical determination of *η*

We have tried also to determine *η *by a theoretical approach. More precisely, we have simulated unknown subtypes by excluding a subtype from the data based on which the emission probabilities of pHMM U were estimated. We then have chosen *η *such that the emission frequencies of the excluded subtype is estimated best (with respect to maximum likelihood). Unfortunately, this approach has filed to values of *η *smaller by an entire order of magnitude than the values found by means of the calibration described above. Consequently, we refrain from using this theoretical approach.

### SIV sequences and Subtype U sequences

In order to check whether USF correctly classifies very divergent sequences, we have applied it to five full-length SIV genomes (AF103818, DQ373063, EF394356, U42720, X52154) from different parts of the SIV clade. As before, we did not allow for assigning subtypes H, J, and K in the subtype classification. In the same way we have tested the 8 full-length Subtype U genomes (AF286236, AF457101, AY046058, EF029066, EF029067, EF029068, EF029069, FJ388921). Except the Subtype U sequence AY046058, all sequences have been correctly identified as completely unknown (about 8% of the genome have not been classified as unknown).

### Comparison with the BI

The BI is a method based on distance and phylogeny. It determines which parts of a query sequence should be classified among known sequences. Moving along the genome of a query sequence with a sliding window, the BI computes a ratio quantifying how closely the query sequence clusters with a subtype clade. On the basis of this quantity, it determines whether the respective part of a query sequence is unclassifiable with respect to the known subtypes.

We apply the BI to a subset of *T_spec_*, as well as the SIV and Subtype U sequences used in the evaluation described in the subsection 'Results - SIV sequences and Subtype U sequences'. As we had to carry out the testing manually, using the web interface of the BI [28], we had to confine ourselves to a limited number of sequences from *T_spec _*and could not test the BI on *T_sens _*at all. (For the purpose of the latter, it would have been necessary to reestimate the parameters of the BI after having removed a subtype from the training data. That, however, the web interface available does not allow.)

Application of the BI to the 5 SIV sequences and the 8 Subtype U sequences from the subsection 'Results - SIV sequences and Subtype U sequences' yields valid results in 3 and 4 cases, respectively. Out of these 7 sequences, all but one Subtype U sequence (AY046058) are classified correctly as completely unknown, with about 6% of the genome of AY046058 being misclassified.

**Figure 10:**
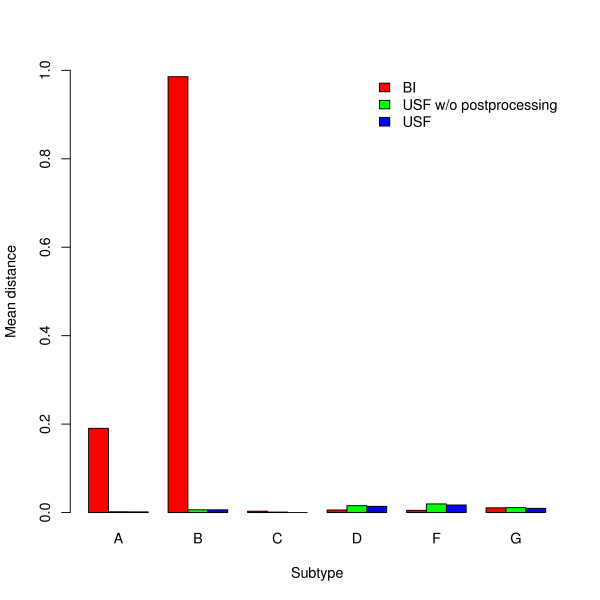
**Comparison of USF and BI**. The mean performance (measured in terms of the fraction of misclassified positions) for 12 random sequences of each subtype for the BI and USF, stratified by the assigned subtype. For USF the results with all segments of length less than 100 bps (green) and without such a removal (green) are displayed.

Testing the BI on 12 sequences for each subtype from *T_spec_*, yield the results illustrated in Figure 10. Since USF tends to misclassify very short segments as unknown for some subtypes, we also compare the BI with USF, removing all segments of length smaller than 100 bps from the outcome of USF.

Using the two-sided Wilcoxon signed-rank test, the version of USF without postprocessing performs significantly better (with respect to our position-wise measure) than the BI for the subtypes A and B. For the Subtype F, the BI is significantly better than USF (*p *= 0.05). For the other subtypes, this test does not yield significant results. If USF is used in the version equipped with postprocessing, it yields significantly better results than the BI for the subtypes A and B, with the differences on the other subtypes being highly insignificant.

### Running time

Excluding the running time of ClustalW and jpHMM (described in [18,17]), the running time for a full length HIV-1 sequence is about 35 seconds on a Linux PC with 3 GHz and 4 GB RAM.

## Discussion and conclusions

We have presented USF, a tool for detection of unclassifiable segments in viral sequences. Using a probabilistic, model-driven approach, the tool is suitable in principle for all species (or other taxa) which are subdivided into subfamilies i) without too many indels separating the subfamilies and ii) where the phylogenetic distances between the subfamilies are not too inhomogeneous.

### Testing

We have applied USF to i) artificial recombinants of two subtypes (excluding one subtype from the training data to simulate an unknown subtype), ii) sequences designated (in the LANL HIV database) as originating from a pure subtype, iii) SIV sequences, and iv) Subtype U sequences. As far as feasible, we have compared our results with the only other tool available with the same scope, the Branching Index (BI).

### Performance of USF

Analyzing the performance of USF by subtype, one can see that it performs considerably better (with respect to specificity) on the subtypes A-C than on D, F, and G, whereas it does not yield acceptable results for the subtypes H, J, and K. Its unsatisfactory performance on the last three subtypes does not come unexpectedly: The subtypes H, J, and K are composed of only 2 or 3 complete genome sequences, and that does not allow for a realistic modelling of the emission probabilities of a pHMM without using an information sharing protocol (see [23]). The weaker performance for subtypes D, F, and G might also be explicable by this effect, with the situation being obfuscated for the Subtype F by the fact that this subtype is divided into two subsubtypes.

The results of the application of USF to artificial recombinants can be explained in part also by the size of the involved subtypes: The poorest results are achieved when subtypes G or J, which both belong to the subtypes of smaller size, act as base subtype. Obviously, the size of the insertion subtype should not have any impact on the performance of USF (and the results also do not suggest that). Astonishingly, there does not seem to be a correlation between the phylogenetic distance of a pair of base and insertion subtypes and the performance of USF on the respective pair: Testing *T_sens _*involves 46 pairs of subtypes. Considering the 13 pairs with the lowest phylogenetic distance, none of them is among the 3 poorest performing pairs and 3 are among the 7 poorest performing. As we have observed a poor performance of USF when the subtypes B and D are the base and insertion subtypes, we may conclude that, if the phylogenetic distance of the subtype pair is above a certain threshold, the performance of USF does not seem to depend on how remotely the subtypes are related exactly.

### Specificity of USF & BI

Comparing USF (employing the removal of very short segments in the outcome) with the BI with respect to specificity, USF, roughly speaking, performs better on some of the large size subtypes (A and B), whereas there are no significant differences on the large size Subtype C and the smaller size subtypes D, F, and G.

### Sensitivity of USF & BI

For a comparison of the sensitivities we had to restrict ourselves to the SIV and Subtype U sequences. In spite of the importance of the sensitivity to assess the performance of USF and BI, the analysis of this characteristic had to be carried out on quite a small test set, due to technical limitations in the implementation of the BI. Except for the Subtype U sequence AY046058, all SIV and Subtype U sequences were classified as unknown by USF as well as the BI. Since both tools detect the same sequence as not completely unknown (although different segments were detected as known), this might be a hint that the classification of AY046058 as a pure Subtype U sequence is questionable. To conclude, our analysis does not reveal any significant differences between USF and the BI with respect to their sensitivity.

### Versatility of USF & BI

With respect to versatility, the BI seems to be slightly inferior to USF (at least in their current versions). As it is not possible to determine *η *by a theoretical approach (as described in paragraph 'Results - Calibration - Theoretical determination of *η*'), both methods require a parameter calibration on training data when applied to a new species, respectively taxon. Regarding breakpoint positions, the BI only provides a graph from which the user would have to deduce the breakpoint positions by visual inspection. Hence, it is not possible to run any automated procedures on the BI if breakpoint positions are required.

### Outlook

In the near future, we plan to incorporate our method in the jpHMM. This would lead to a tool capable not only of assigning the known subtypes of HIV-1 (or subfamilies of other viruses or species) to a query sequence (or parts of it) but also of detecting segments of the genome stemming from a subtype yet unknown. Moreover, we are currently working on the implementation of an information sharing protocol for the jpHMM, which then would attenuate the poor performance of USF when applied to the small size subtypes.

In addition, it has been discussed whether the core gene of some D/E-recombinants of Hepatitis B virus (HBV) might stem from a clade which became rare or extinct [29]. We will apply USF to HBV data in order to investigate this question.

Furthermore, it has been claimed that the HBV genotype G is a recombinant between i) an ancestor comparable in divergence to those between the genotypes A-E, contributing the S gene, and ii) an HBV variant which is much more divergent, contributing the rest of the genome [30]. In the face of this finding, we plan to incorporate more than one unknown subtype in our model so that different degrees of divergence can be modelled.

### Miscellaneous

As mentioned in the section 'Background', it is not possible to find unknown sequence segments by identifying regions of small a posterior probabilities for all of the known subfamilies when applying the jpHMM for example. That is easily exemplified as follows: Let us assume there were only two subtypes A and B and we examined a sequence stemming from an unknown subtype which is genetically considerably closer to Subtype A than to Subtype B. Then this sequence would achieve very large a posteriori probabilities for Subtype A and very small ones for Subtype B. Thus, it would falsely be classified as known.

USF is implemented in C++ and the source code is freely available (see additional file 1).

## Authors' contributions

TU implemented and validated the algorithm. AKS carried out modifications on jpHMM. JB provided statistical expertise. MS and BM guided the project, MS contributed to the model development. IB conceived the approach, developed, implemented and tested the algorithm and supervised the program development. All authors read and approved the final manuscript.

## Supplementary Material

Additional file 1 **Source code**. C++ implementation of USF. Click here for file

## References

[B1] Korber B Gaschen B Yusim K Thakallapally R Kesmir C Detours V Evolutionary and immunological implications of contemporary HIV-1 variation Br Med Bull 2001 58 19 42 10.1093/bmb/58.1.19 11714622

[B2] Leitner T The molecular epidemiology of human viruses 2002 Springer Berlin

[B3] Hraber P Fischer W Bruno W Leitner T Kuiken C Comparative analysis of hepatitis C virus phylogenies from coding and non-coding regions: the 5' untranslated region (UTR) fails to classify subtypes Virology Journal 2006 3 103 10.1186/1743-422X-3-103 17169155 PMC1764733

[B4] Rhodes T Wargo H Hu WS High Rates of Human Immunodeficiency Virus Type 1 Recombination: Near-Random Segregation of Markers One Kilobase Apart in One Round of Viral Replication J Virol 2003 77 20 11193 11200 10.1128/JVI.77.20.11193-11200.2003 14512567 PMC224990

[B5] Hahn BH Shaw GM De KM Sharp PM AIDS as a Zoonosis: Scientific and Public Health Implications Science 2000 287 5453 607 614 10.1126/science.287.5453.607 10649986

[B6] Robertson DL Anderson JP Bradac JA Carr JK Foley B Funkhouser RK Gao F Hahn BH Kalish ML Kuiken C Learn GH Leitner T McCutchan F Osmanov S Peeters M Pieniazek D Salminen M Sharp PM Wolinsky S Korber B HIV-1 nomenclature proposal Science 2000 288 55 57 10.1126/science.288.5463.55d 10766634

[B7] Hoelscher M Dowling WE Sanders-Buell E Carr JK Harris ME Thomschke A Robb ML Birx DL McCutchan FE Detection of HIV-1 subtypes, recombinants, and dual infections in East Africa by a multi-region hybridization assay AIDS 2002 16 2055 2064 10.1097/00002030-200210180-00011 12370505

[B8] LANL HIV Databases: CRFs 2011 Http://www.hiv.lanl.gov/content/sequence/HIV/CRFs/CRFs.html

[B9] de Oliveira T Deforche K Cassol S Salminen M Paraskevis D Seebregts C Snoeck J van Rensburg EJ Wensing AMJ van de Vijver DA Boucher CA Camacho R Vandamme AM An automated genotyping system for analysis of HIV-1 and other microbial sequences Bioinformatics 2005 21 19 3797 3800 10.1093/bioinformatics/bti607 16076886

[B10] Recombinant Identification Program Web Interface http://www.hiv.lanl.gov/content/sequence/RIP/RIP.html

[B11] Zhang M Schultz AK Calef C Kuiken C Leitner T Korber B Morgenstern B Stanke M jpHMM at GOBICS: a web server to detect genomic recombinations in HIV-1 Nucleic Acids Res 2006 34 S2 W463 465 10.1093/nar/gkl255 16845050 PMC1538796

[B12] Schultz AK Zhang M Bulla I Leitner T Korber B Morgenstern B Stanke M jpHMM: Improving the reliability of recombination prediction in HIV-1 Nucl Acids Res 2009 37 Web Server W647 651 10.1093/nar/gkp371 19443440 PMC2703979

[B13] Maydt J Lengauer T Recco: recombination analysis using cost optimization Bioinformatics 2006 22 9 1064 1071 10.1093/bioinformatics/btl057 16488909

[B14] Pandit A Sinha S Using genomic signatures for HIV-1 sub-typing BMC Bioinformatics 2010 11 Suppl 1 S26 10.1186/1471-2105-11-S1-S26 20122198 PMC3009497

[B15] Wilbe K Salminen M Laukkanen T McCutchan F Ray SC Albert J Leitner T Characterization of novel recombinant HIV-1 genomes using the branching index Virology 2003 316 116 125 10.1016/j.virol.2003.08.004 14599796

[B16] Hraber P Kuiken C Waugh M Geer S Bruno WJ Leitner T Classification of hepatitis C virus and human immunodeficiency virus-1 sequences with the branching index J Gen Virol 2008 89 9 2098 2107 10.1099/vir.0.83657-0 18753218 PMC2754793

[B17] Schultz AK Zhang M Leitner T Kuiken C Korber B Morgenstern B Stanke M A jumping profile Hidden Markov Model and applications to recombination sites in HIV and HCV genomes BMC Bioinformatics 2006 7 265 10.1186/1471-2105-7-265 16716226 PMC1525204

[B18] Chenna R Sugawara H Koike T Lopez R Gibson TJ Higgins DG Thompson JD Multiple sequence alignment with the Clustal series of programs Nucl Acids Res 2003 31 13 3497 3500 10.1093/nar/gkg500 12824352 PMC168907

[B19] Krogh A Brown M Mian IS Sjölander K Haussler D Hidden Markov Models in Computational Biology: Applications to Protein Modeling Journal of Molecular Biology 1994 235 5 1501 1531 10.1006/jmbi.1994.1104 8107089

[B20] Eddy S Profile hidden Markov models Bioinformatics 1998 14 9 755 763 10.1093/bioinformatics/14.9.755 9918945

[B21] Viterbi A Error bounds for convolutional codes and an asymptotically optimum decoding algorithm Information Theory, IEEE Transactions 1967 13 2 260 269 10.1109/TIT.1967.1054010

[B22] Sjölander K Karplus K Brown M Hughey R Krogh A Mian I Haussler D Dirichlet mixtures: a method for improved detection of weak but significant protein sequence homology Comput Appl Biosci 1996 12 4 327 345 8902360 10.1093/bioinformatics/12.4.327

[B23] Brown DP Krishnamurthy N Sjölander K Automated Protein Subfamily Identification and Classification PLoS Comput Biol 2007 3 8 e160 10.1371/journal.pcbi.0030160 17708678 PMC1950344

[B24] Akaike H A new look at the statistical model identification Automatic Control, IEEE Transactions 1974 19 6 716 723 10.1109/TAC.1974.1100705

[B25] Price MN Dehal PS Arkin AP FastTree: Computing Large Minimum Evolution Trees with Profiles instead of a Distance Matrix Mol Biol Evol 2009 26 7 1641 1650 10.1093/molbev/msp077 19377059 PMC2693737

[B26] FigTree Http://tree.bio.ed.ac.uk/software/gtree/

[B27] Korber B Foley B Kuiken C Pillai S Sodroski J Numbering Positions in HIV Relative to HXB2CG Human Retroviruses and AIDS 1998, Los Alamos, NM: Theoretical Biology and Biophysics Group, Los Alamos National Laboratory 1998 102 111

[B28] Branching Index Web Interface http://www.hiv.lanl.gov/content/sequence/phyloplace/PhyloPlace.html

[B29] Simmonds P Midgley S Recombination in the Genesis and Evolution of Hepatitis B Virus Genotypes J Virol 2005 79 24 15467 15476 10.1128/JVI.79.24.15467-15476.2005 16306618 PMC1316029

[B30] Kato H Orito E Gish RG Sugauchi F Suzuki S Ueda R Miyakawa Y Mizokami M Characteristics of Hepatitis B Virus Isolates of Genotype G and Their Phylogenetic Differences from the Other Six Genotypes (A through F) J Virol 2002 76 12 6131 6137 10.1128/JVI.76.12.6131-6137.2002 12021346 PMC136184

